# Non-occlusive mesenteric ischemia in critically ill patients

**DOI:** 10.1371/journal.pone.0279196

**Published:** 2022-12-19

**Authors:** Byunghyuk Yu, Ryoung-Eun Ko, Keesang Yoo, Eunmi Gil, Kyoung-Jin Choi, Chi-Min Park

**Affiliations:** 1 Department of Critical Care Medicine, Samsung Medical Center, Sungkyunkwan University School of Medicine, Seoul, Korea; 2 Intensive Care Unit, Kyungpook National University Chilgok Hospital, Daegu, Korea; 3 School of Medicine, Kyungpook National University, Daegu, Korea; 4 Department of Surgery, Samsung Medical Center, Sungkyunkwan University School of Medicine, Seoul, Korea; All India Institute of Medical Sciences, INDIA

## Abstract

**Background:**

Non-occlusive mesenteric ischemia (NOMI) is a life-threatening acute condition that has an overall in-hospital mortality rate of up to 75%. Critically ill patients are often admitted to intensive care units (ICUs) due to shock, and these patients are frequently at risk of developing NOMI. The objective of this study was to determine the clinical features of critically ill patients with NOMI and evaluate the risk factors for in-hospital mortality among these patients.

**Methods:**

We reviewed the electronic medical records of 7,346 patients who underwent abdominal contrast-enhanced computed tomography during their ICU stay at Samsung Medical Center (Seoul, Korea) between January 1, 2010 and December 31, 2019. After reviewing each patient’s computed tomography (CT) scans, 60 patients were diagnosed with NOMI and included in this analysis. The patients were divided into survivor (n = 23) and non-survivor (n = 37) groups according to the in-hospital mortality.

**Results:**

The overall sequential organ failure assessment (SOFA) score for the included patients upon admission to the ICU was 8.6 ± 3.1, and medical ICU admissions were most common (66.7%) among the patients. The SOFA score upon admission to the ICU was higher for the non-survivors than for the survivors (9.4 vs. 7.4; p = 0.017). Non-survivors were more often observed in the medical ICU admissions (39.1% vs. 83.8%) than in the surgical ICU admissions (47.8% vs. 10.8%) or the cardiac ICU admissions (13.0% vs. 5.4%). Laboratory test results, abdominal CT findings, and the use of vasopressors and inotropes did not differ between the two groups. In a multivariable analysis, SOFA scores >8 upon admission to the ICU (odds ratio [OR] 4.51; 95% 1.12–18.13; p = 0.034), patients admitted to the ICU with medical problems (OR 7.99; 95% 1.73–36.94; p = 0.008), and abdominal pain (OR 4.26; 95% 1.05–17.35; p = 0.043) were significant prognostic predictors for in-hospital mortality.

**Conclusions:**

The SOFA score >8 upon admission to the ICU, admission to the ICU for medical problems, and abdominal pain at diagnosis are associated with increased mortality among patients with NOMI.

## Introduction

Non-occlusive mesenteric ischemia (NOMI), which is an acute form of mesenteric ischemia [1], is characterized by the absence of an embolic or thrombotic occlusion of the mesenteric vessels and the functional vasoconstriction of the splanchnic arterial vessels [2]. NOMI accounts for up to 15% of acute mesenteric ischemia cases, and the overall in-hospital mortality among patients with NOMI is 21–75% [2, 3]. Coronary heart disease and hypovolemic, septic, and cardiogenic shock, all of which are common among patients in intensive care units (ICUs), are known risk factors for the development of NOMI [4–6]. Therefore, patients in the ICU are frequently at risk of developing NOMI.

Diagnosing NOMI in the ICU remains challenging because the clinical symptoms of the disorder are not specific, and patients in the ICU are often sedated and unable to describe gastrointestinal symptoms. Consequently, most cases of NOMI are diagnosed after it has reached an advanced state, which results in treatment delays and poor outcomes. Abdominal contrast-enhanced computed tomography (CT) is useful for diagnosing NOMI [7, 8].

Selective angiography has long been regarded as the gold standard for diagnosing NOMI [9]. However, abdominal contrast-enhanced CT has also gained recognition for its essential role in the diagnosis of the disorder [8, 10, 11], making it an important diagnostic modality for clinicians to use when developing clinical management strategies for patients with NOMI, such as whether and when to perform selective angiography or surgical intervention.

Therefore, the aim of this study was to investigate the clinical characteristics of critically ill patients with NOMI and evaluate the risk factors for in-hospital mortality.

## Materials and methods

### Study population

**Fig 1:**
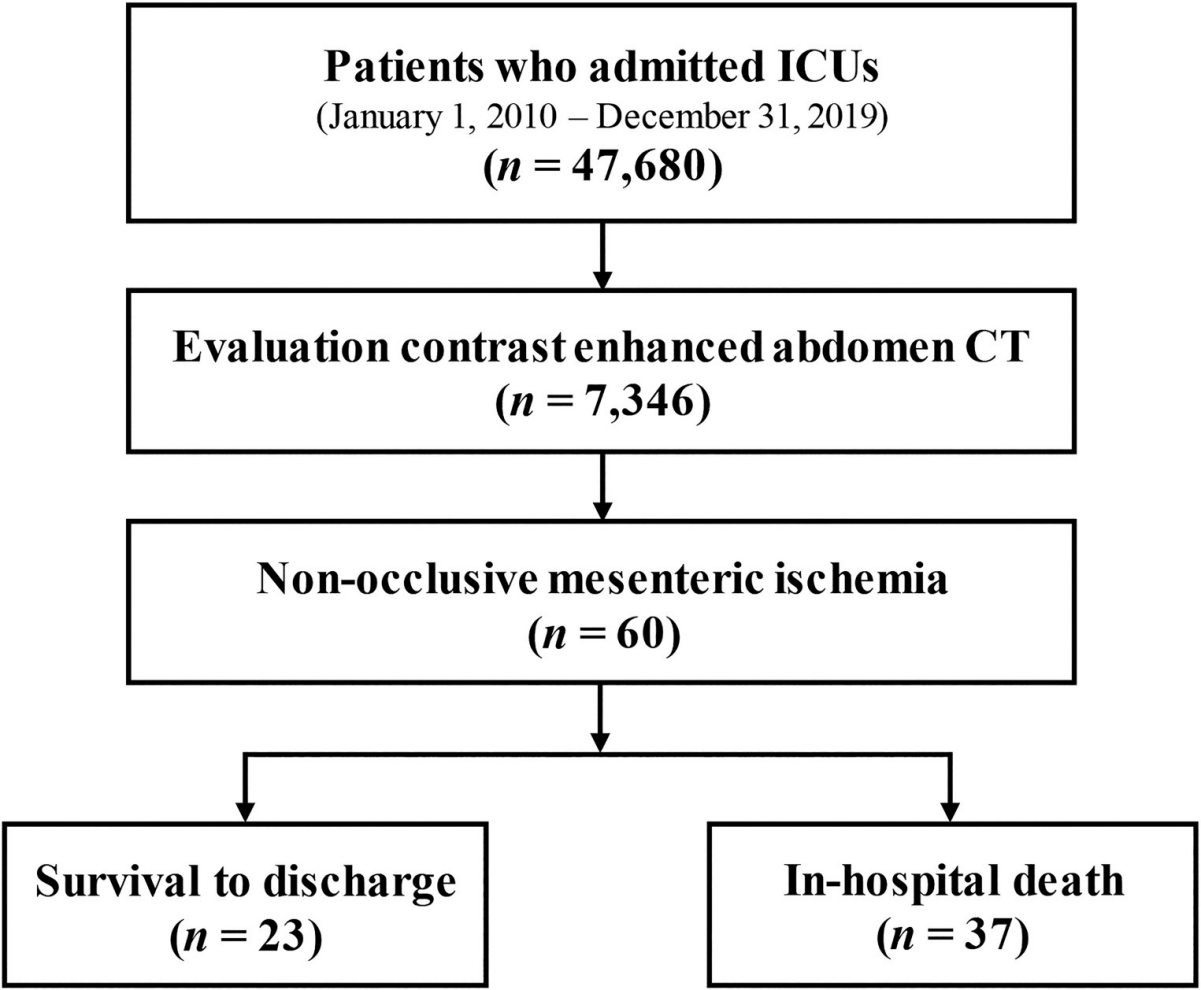
The patient enrollment scheme for this study. CT, computed tomography; ICU, intensive care unit.

We conducted a retrospective cohort study of all the patients aged ≥18 years who were admitted to the ICUs at Samsung Medical Center, which is a 1,989-bed university-affiliated tertiary referral hospital in Seoul, Korea, between January 1, 2010 and December 31, 2019. A total of 47,680 patients were admitted to the ICUs in the facility during the study period, and 7,436 of these patients were evaluated during admission using abdominal contrast-enhanced CT. NOMI was defined in the study as follows: (1) clinical suspicion; (2) radiographic signs (such as vasospasm of the superior mesenteric artery (SMA) and its branches, as indicated by a reduced vessel diameter and contrast) on abdominal contrast-enhanced CT; and (3) exclusion of thrombotic or embolic mesenteric artery occlusions [12]. A total of 60 patients met these criteria during the study period. The eligible patients were divided into survivor and non-survivor groups according to the hospital mortality (Fig 1). The Institutional Review Board of Samsung Medical Center approved this study and waived the requirement for informed consent due to the observational nature of the investigation. Additionally, the patients’ information was anonymized and de-identified before the analysis began.

### Data collection

The clinical, laboratory, and outcome data for the patients were retrospectively analyzed by reviewing their electronic medical records. Demographic data, including age, sex, sequential organ failure assessment (SOFA) score, admission route, identity of the ICU to which the patient was admitted, diagnosis, and comorbidities were recorded upon each patient’s admission to the ICU. To compare the clinical signs of NOMI with the in-hospital mortality, we collected the vital sign data, laboratory test results, and signs on abdominal contrast-enhanced CT that were obtained for each patient at the time of the NOMI diagnosis. In this study, we defined the time of the NOMI diagnosis as the time when the abdominal contrast-enhanced CT images were evaluated. The CT results were read by two radiologists, and three intensivists who independently reviewed all the abdominal contrast-enhanced CT images using a viewing console made the final diagnosis decisions by consensus. The abdominal contrast-enhanced CT images were assessed for small bowel ischemia, pneumatosis intestinalis, NOMI, ischemic colitis, and SMA size. The vasopressor and inotrope initiation and discontinuation times, dose adjustments for each vasopressor and inotrope (in mcg/kg/min, except for vasopressin, which was in units/min), and the input and output balances for each patient were recorded by the bedside nurse. The NOMI management methods were also collected.

### Statistical analysis

The categorical data are presented as numbers and percentages, and the continuous variables are presented as medians with interquartile ranges or means with standard deviations. To compare the characteristics and clinical outcomes between the two groups, we used χ^2^ tests or Fisher’s exact tests for the categorical variables (when applicable) and Mann–Whitney U tests for the continuous variables. A two-tailed test with a p-value <0.05 was considered statistically significant for all the analyses. A logistic regression analysis was performed to identify the predictors of in-hospital mortality. Age, sex, and variables that appeared to be related in the univariate analysis (those with a p-value <0.2) were analyzed further using multivariable regression models. Each variable’s odds ratio (OR) is reported with the 95% confidence interval (CI). All the data analyses were performed using R Statistical Software (version 3.2.5; R Foundation for Statistical Computing, Vienna, Austria).

## Results

### Patient characteristics

The baseline clinical characteristics of the study cohort are shown in Table 1. The mean age of the patients was 61.6 ± 14.1 years, and 63.3% of the patients were male. The in-hospital mortality was 62.3%, and the SOFA score upon admission to the ICU was 8.6 ± 3.1. Most of the patients were admitted to the ICU from the general ward (48.3%), followed by the emergency room (35.0%). While most of the patients were admitted to medical ICUs (66.7%), 25.0% were admitted to surgical ICUs, and 8.3% were admitted to the cardiac ICU. Shock (40.0%), post-surgery complications (26.7%), and respiratory failure (21.7%) were the most frequent concerns at admission. The comorbidities of the survivors were similar to those of the non-survivors, and diabetes (41.7%) and chronic kidney disease (31.7%) were the most common comorbidities among the included patients.

**Table 1:** Baseline characteristics of the study population.

Variables	Survivors (n = 23)	Non-survivors (n = 37)	p
Age (years)	61.6±12.3	61.6±15.4	0.997
Sex (males)	13 (56.5)	25 (67.6)	0.557
SOFA score upon admission to the ICU	7.4±2.8	9.4±3.0	0.017
Admission route			0.888
General ward	12 (52.2)	17 (45.9)	
Emergency room	7 (30.4)	14 (37.8)	
ICU transfer	4 (17.4)	5 (13.5)	
Outpatient	0 (0.0)	1 (2.7)	
Department			0.001
Medical	9 (39.1)	31 (83.8)	
Surgical	11 (47.8)	4 (10.8)	
Cardiac	3 (13.0)	2 (5.4)	
Diagnosis upon admission to the ICU			0.083
Shock	8 (34.8)	16 (43.2)	
Respiratory failure	4 (17.4)	9 (24.3)	
Surgical	10 (43.5)	6 (16.2)	
Neurologic	0 (0.0)	5 (13.5)	
Renal failure	1 (4.3)	1 (2.7)	
Comorbidity			
Myocardial infarction	5 (21.7)	4 (10.8)	0.284
Congestive heart failure	3 (13.0)	5 (13.5)	1.000
Peripheral vascular disease	1 (4.3)	1 (2.6)	1.000
Cerebral vascular disease	5 (21.7)	7 (18.9)	1.000
Chronic pulmonary disease	2 (8.7)	6 (16.2)	0.698
Peptic ulcer	1 (4.3)	0 (0.0)	0.383
Chronic liver disease	4 (17.4)	9 (23.7)	0.749
Diabetes mellitus	11 (47.8)	14 (37.8)	0.622
Hemiplegia	1 (4.3)	3 (8.1)	1.000
Chronic kidney disease	8 (34.8)	11 (29.7)	0.902
Malignancy			0.311
Hematology	3 (13.0)	11 (29.7)	
Oncology	4 (17.4)	7 (18.9)	

The values are presented as means ± standard deviations or numbers with percentages in parentheses.

ICU, intensive care unit; SOFA, sequential organ failure assessment.

The SOFA score upon admission to the ICU was higher for the non-survivors than for the survivors (9.4 vs. 7.4; p = 0.017). Non-survivors were more often observed in the medical ICU admissions (39.1% vs. 83.8%) than in the surgical ICU admissions (47.8% vs. 10.8%) or the cardiac ICU admissions (13.0% vs. 5.4%).

### Clinical characteristics related to NOMI

**Fig 2:**
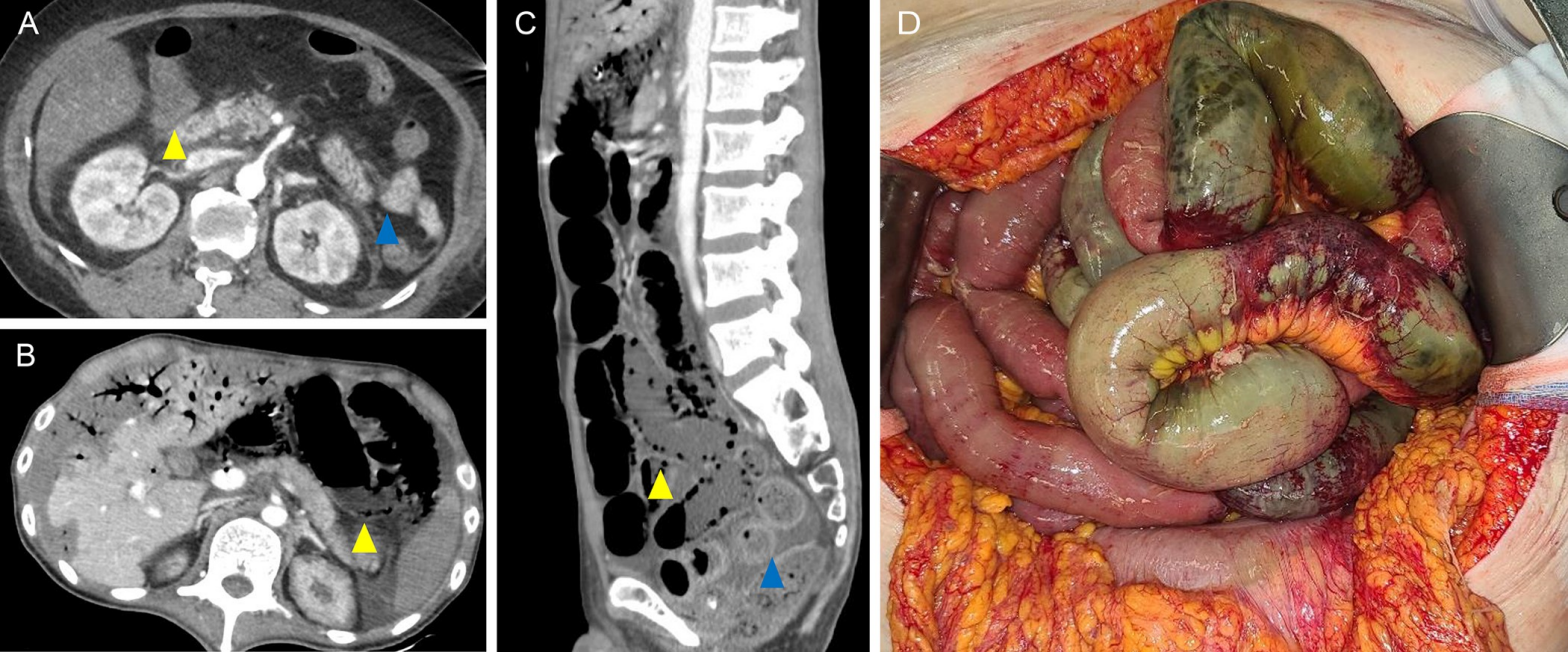
Contrast-enhanced CT images and intraoperative bowel images of non-occlusive mesenteric ischemia. (A) Contract-enhanced abdomen CT images of survivor, transverse image (B) Contract-enhanced abdomen CT images of non-survivor, transverse image (C) Contract-enhanced abdomen CT images of non-survivor, sagittal image (D) intraoperative bowel images; multiple segmental bowel necrosis was observed, but also normal bowel and mesentery found between necrotic bowel segments. Yellow arrows indicate the dilated and thinned bowel and blue arrows indicate normal bowel.

The clinical symptoms of the non-survivors were similar to those of the survivors, except for the presence of abdominal pain (62.2% vs. 26.1%, respectively; p = 0.014) (Table 2). No significant differences in the vital signs were observed between the groups. In addition, the survivors and non-survivors did not show significant differences in the results from the laboratory tests, which included C-reactive protein (10.0 mg/dl vs. 10.6 mg/dl; p = 0.294), lactate dehydrogenase (854 IU/L vs. 854 IU/L; p = 238), and lactate (2.1 mmol/L vs. 2.4 mmol/L; p = 0.204). Small bowel ischemia (88.3%) and ischemic colitis (68.3%) were often observed in the abdominal contrast-enhanced CT findings for all the patients. Of the 53 patients with small bowel ischemia on abdominal CT, 3 presented in the duodenum, 18 presented in the jejunum, 23 presented in the ileum, and 9 presented in the jejunum to ileum. The SMA sizes (6.3 mm [survivors] vs. 5.9 mm [non-survivors]; p = 0.474) were not significantly different between the groups. Contrast-enhanced CT images of patients with NOMI (A—C) and intraoperative ischemic bowel image (D) were presented in Fig 2. Among all the patients, 10% underwent surgical interventions, including small bowel resection for three patients, small bowel resection and a right hemicolectomy for one patient, and a total colectomy for two patients. The remaining patients received optimal medical treatment for NOMI.

**Table 2:** Characteristics of the patients with NOMI.

Variables	Survivors (n = 23)	Non-survivors (n = 37)	p
Symptom at diagnosis			
Abdominal pain	6 (26.1)	23 (62.2)	0.014
Diarrhea	9 (39.1)	10 (27.0)	0.487
Hematochezia	15 (21.7)	10 (26.3)	0.336
Suspected diagnosis upon imaging			0.247
Ischemic	6 (26.1)	16 (43.2)	
Infectious	7 (30.4)	12 (32.4)	
Others	10 (43.5)	9 (24.3)	
Vital signs at diagnosis			
SpO_2_ (%)	100.0 (99.0–100.0)	99.0 (98.0–100.0)	0.270
Respiratory rate/min	20.0 (17.5–27.5)	19.0 (17.0–24.0)	0.450
Heart rate/min	97.6±26.8	106.4±16.0	0.167
Systolic blood pressure (mmHg)	113.1±19.7	108.6±22.4	0.429
Diastolic blood pressure (mmHg)	65.3±21.2	62.3±13.1	0.549
Mean arterial pressure (mmHg)	75.1±19.0	73.7±13.1	0.756
Body temperature (°C)	37.0±1.3	36.8±0.9	0.456
Laboratory test results at diagnosis			
White blood cells (10^3^/L)	11.4 (6.7–15.2)	7.7 (0.8–13.8)	0.070
Hemoglobin (g/dl)	9.1 (8.0–10.1)	9.0 (7.9–11.0)	0.855
Platelet count (10^3^/L)	59.0 (45.5–109.0)	45.0 (27.0–94.0)	0.102
Total bilirubin (mg/dl)	1.3 (0.9–2.5)	2.1 (1.0–6.3)	0.238
AST (U/L)	52.0 (27.0–168.0)	63.0 (47.0–146.0)	0.346
ALT (U/L)	45.0 (26.0–118.0)	47.0 (24.0–153.0)	0.879
BUN (mg/dl)	32.9 (25.6–44.2)	38.6 (21.5–60.1)	0.277
Creatinine (mg/dl)	1.4 (1.2–2.0)	1.6 (0.9–2.3)	0.855
C-reactive protein (mg/dl)	10.0 (4.7–12.3)	10.6 (5.9–20.1)	0.294
Lactate dehydrogenase (IU/L)	854 (854–960)	854 (786–854)	0.238
Lactate (mmol/L)	2.1 (1.5–3.8)	2.4 (1.8–5.5)	0.204
Abdominal CT signs			
Small bowel ischemia	20 (87.0)	33 (89.2)	1.000
Pneumatosis intestinalis	1 (4.3)	7 (18.9)	0.138
NOMI	3 (13.0)	12 (32.4)	0.168
Ischemic colitis	17 (73.9)	25 (65.8)	0.655
SMA size (mm)	6.3±1.7	5.9±1.7	0.474
Surgical intervention	1 (4.3)	5 (13.5)	0.391
Small bowel resection	1 (100.0)	2 (40.0)	
Small bowel resection and right hemicolectomy	0 (0.0)	1 (20.0)	
Total proctocolectomy	0 (0.0)	2 (40.0)	

The values are presented as medians with interquartile ranges in parentheses, means ± standard deviations, or numbers with percentages in parentheses.

AST, aspartate aminotransferase; ALT, alanine aminotransferase; BUN, blood urea nitrogen; CT, computed tomography; NOMI, non-occlusive mesenteric ischemia; SMA, superior mesenteric artery.

### Vasopressors and inotropes

**Fig 3:**
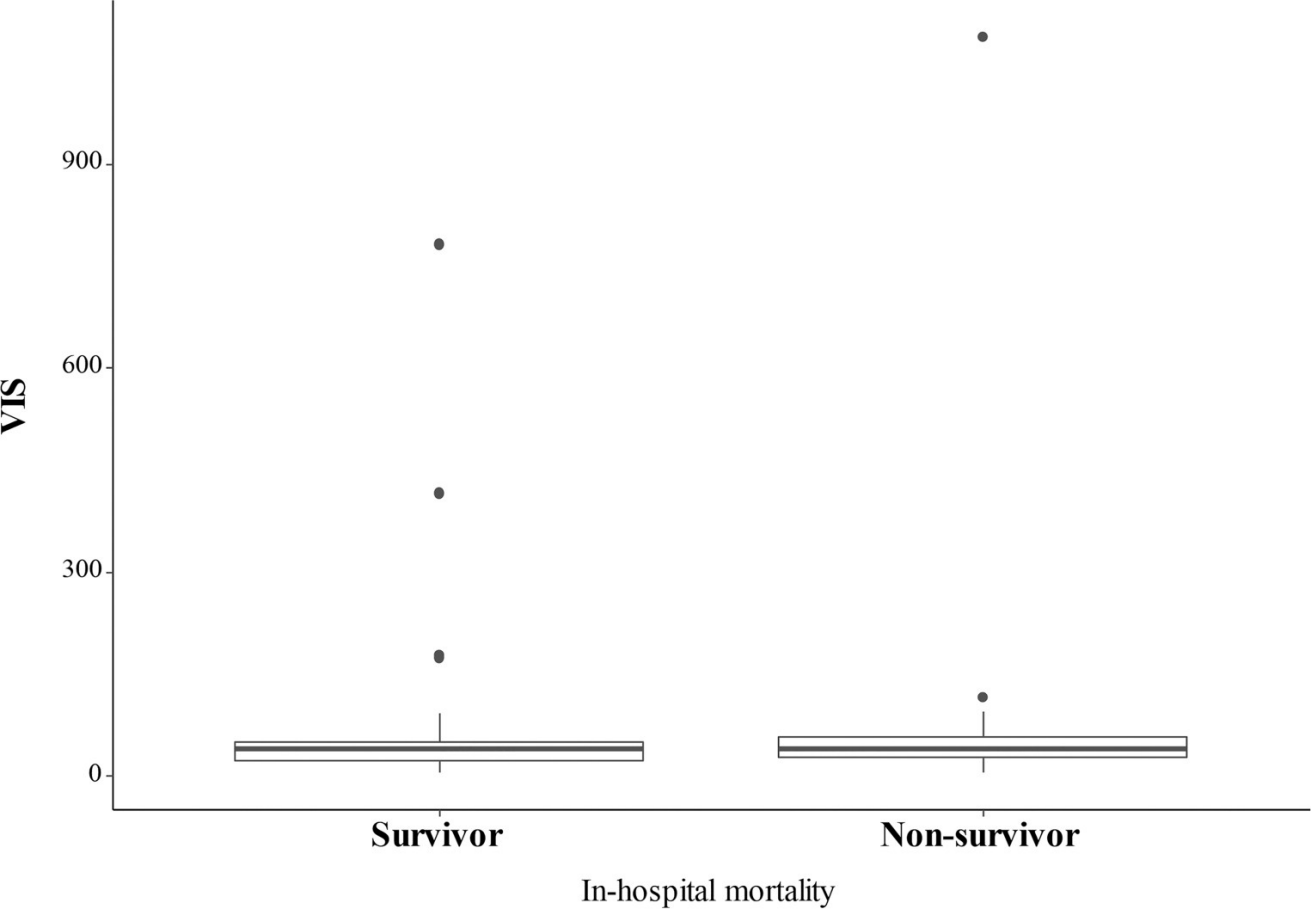
The peak vasoactive-inotropic scores according to the in-hospital mortality. The bold line in the middle indicates the median, and the top and bottom of the square indicate the interquartile ranges of the vasoactive-inotropic scores. VIS, vasoactive-inotropic score.

The duration and mean dose of the vasopressors and inotropes used for each patient were evaluated prior to the NOMI diagnosis being made (Table 3). Norepinephrine (81.7%) was most often administered to the included patients, followed by vasopressin (40.0%). There were no significant differences in the drug durations or dosages between the groups. There was also no difference in the peak vasoactive-inotropic scores (40 [5.0–783.0] vs. 40 [5.0–1,088.0], p = 0.635) between the groups (Fig 3). Among the included patients, 45% received more than two vasopressors or inotropes. The input and output balances for the survivors and non-survivors were 22,912 ml and 18,261 ml, respectively (p = 0.726).

**Table 3:** Vasopressor and inotrope administration prior to the NOMI diagnosis.

Variables	Survivors (n = 23)	Non-survivors (n = 37)	p
Between vasopressor and inotrope initiation and diagnosis, day	2.8 (0.6–8.1)	6.4 (2.6–13.1)	0.154
Norepinephrine	17 (73.9)	32 (86.5)	0.306
Duration (min)	1,414 (603–2,842)	1,785 (598–4,719)	0.512
μg/kg/min	0.1 (0.1–0.2)	0.2 (0.1–0.3)	0.322
Vasopressin	6 (26.1)	18 (48.6)	0.143
Duration (min)	793 (381–3,260)	1,600 (330–3,696)	0.868
U/min	0.01 (0.01–0.03)	0.01 (0.01–0.03)	0.972
Dopamine	3 (13.0)	1 (2.7)	0.153
Duration (min)	2,141 (1,101–3,609)	3,809 (3,809–3,809)	
μg/kg/min	6.3 (5.7–7.2)	4.7 (4.7–4.7)	
Dobutamine	4 (17.4)	3 (8.1)	0.412
Duration (min)	1,901 ± 3,597	10,364 ± 10,077	0.172
μg/kg/min	6.3 ± 2.0	6.3 ±3.4	0.973
Epinephrine	4 (17.4)	3 (8.1)	0.412
Duration (min)	785 ± 1,046	33 ± 32.8	0.246
μg/kg/min	0.1 (0.0–4.0)	0.1 (0.1–5.4)	0.400
More than two drugs	8 (34.8)	19 (51.4)	0.323
Input and output balances before diagnosis (ml)	22,912 (6,945–40,430)	18,261 (10,315–34,492)	0.726

The values are presented as medians with interquartile ranges in parentheses, means ± standard deviations, or numbers with percentages in parentheses.

NOMI, non-occlusive mesenteric ischemia.

### Predictors of in-hospital mortality

The multivariable logistic regression analysis revealed that a SOFA score >8 upon admission to the ICU (OR 4.51; 95% 1.12–18.13; p = 0.034), patients admitted to the ICU with medical problems (OR 7.99; 95% 1.73–36.94; p = 0.008), and abdominal pain (OR 4.26; 95% 1.05–17.35; p = 0.043) were significant prognostic predictors for in-hospital mortality (Table 4).

**Table 4:** In-hospital mortality predictors for the patients with NOMI.

	Univariable			Multivariable		
	OR	95% CI	p	Adjusted OR	95% CI	p
Age (years)	1.00	0.96–1.04	0.997	1.03	0.98–1.08	0.234
Sex (females)	0.62	0.21–1.83	0.389	0.50	0.13–1.96	0.317
SOFA >8*	3.46	1.16–10.31	0.026	4.51	1.12–18.13	0.034
Department**						
Medical	9.47	2.42–37.05	0.001	7.99	1.73–36.94	0.008
Cardiac	1.83	0.22–15.33	0.576	1.27	0.12–13.98	0.844
Abdominal pain	4.65	1.48–14.61	0.008	4.26	1.05–17.35	0.043

*The reference group had a SOFA score ≤8.

**The reference group consisted of patients in surgical ICUs.

ICU, intensive care unit; SOFA, sequential organ failure assessment; OR, odds ratio; CI, confidence interval.

## Discussion

In this retrospective cohort study, we evaluated the clinical, laboratory, imaging, and vasopressor and inotrope usage characteristics and the in-hospital mortality among patients with NOMI. Our findings suggest that a poor prognosis is associated with high SOFA scores, abdominal symptoms, and patients with NOMI who have been admitted to medical ICUs. Vasopressor and inotrope use, SMA size, and laboratory results, all of which have previously been shown to be risk and prognostic factors for NOMI, were not significant predictors of in-hospital mortality among the patients with NOMI in the present study.

Within the study cohort, the non-survivor group had a higher SOFA score at the time of the NOMI diagnosis than did the survivor group. Organ failure is a dynamic process, and the degree of dysfunction may vary over time [13, 14]. The SOFA score, which is widely used for describing organ dysfunction in critically ill patients, is composed of scores from six organ systems, and each score ranges from 0–4 according to the degree of dysfunction [14, 15]. Previous studies have shown that a high SOFA score is an independent risk and prognostic factor for NOMI [16, 17]. In a retrospective case-control study of severely burned patients, Soussi et al. evaluated potentially modifiable risk factors for the development of NOMI and found that a decreased cardiac index within the first 24 h and a higher SOFA score on day one were associated with the development of NOMI [16]. Murata et al. evaluated prognostic factors for NOMI among 44 patients with the disorder [17], and their results revealed that a SOFA score ≥10 was associated with increased mortality. We obtained similar results in the present study, as a SOFA score >8 was shown to be a significant prognostic factor for in-hospital mortality among the patients with NOMI. Therefore, careful management of NOMI is required for patients with multi-organ failure.

Medical patients are often admitted to the ICU for management of septic or hypovolemic shock or respiratory failure. Although recent clinical practice guidelines recommend light sedation for patients in the ICU, some patients require deep sedation during the initial phase of ICU management [18, 19]. Therefore, for medical ICU patients, early evaluation of abdominal symptoms might be difficult. In addition, physicians often overlook the monitoring of abdominal symptoms in medical patients, which can lead to a delayed NOMI diagnosis and increase in mortality. However, NOMI often presents with vague symptoms [20]. In the present study, abdominal pain was found to be an independent predictor of in-hospital mortality among the patients with NOMI. Taken together, the results of this study provide important insights into the monitoring of abdominal symptoms in medical ICU patients.

Several reports have suggested that vasopressors and inotropes are associated with the development of NOMI [21, 22]. Critically ill patients with hemodynamic instability are often at the highest risk of developing NOMI. For these patients, medications commonly used during resuscitation or major surgery have been identified as risk factors for NOMI development [23]. Notably, vasopressin has been shown to reduce intestinal blood flow and impair microvascular flow via vasoconstriction [24]. Norepinephrine, which is the first choice for treating septic shock, also increases global splanchnic oxygen extraction [25]. Although these medications affect the development of NOMI, the dose and duration of the vasopressors and inotropes used in the present study did not affect the mortality outcomes for the patients with NOMI. Interestingly, some reports have shown that vasopressin administration during the treatment of NOMI improves intestinal perfusion and hospital survival [26, 27]. Therefore, for patients who have already developed NOMI, clinicians may find it helpful to maintain bowel perfusion with appropriate inotropes and vasopressors. Further studies are needed to fully understand the effects of inotropes and vasopressors on NOMI.

Although the results of this study provide additional information on patients with NOMI, there are potential limitations that should be acknowledged. First, because this was a retrospective cohort study, there was a potential risk of confounding and bias. Second, the NOMI diagnoses were based on solely abdominal contrast-enhanced CT findings, rather than those from selective angiography, endoscopy or laparoscopy, and we did not routinely perform additional selective angiography or continuous arterial vasodilator infusion. This bias may have important consequences.

## Conclusion

This study was designed to determine the clinical factors that are associated with in-hospital mortality for critically ill patients with NOMI. The findings from this study suggest that a SOFA score >8 upon admission to the ICU, admission to the ICU for medical problems, and abdominal pain at the time of the diagnosis are associated with increased mortality. These results highlight the importance of closely monitoring abdominal symptoms in patients who are admitted to the ICU. Further investigation of NOMI in ICU patients is strongly recommended.

## List of abbreviations

NOMI: non-occlusive mesenteric ischemia
ICU: intensive care unit
CT: computed tomography
SMA: superior mesenteric artery
OR: odds ratio
CI: confidence interval
